# High levels of genetic diversity in *Penaeus monodon* populations from the east coast of India

**DOI:** 10.1186/2193-1801-2-671

**Published:** 2013-12-13

**Authors:** Gulab Dattarao Khedkar, A. Chandrashekar Reddy, Tetszuan Benny Ron, David Haymer

**Affiliations:** Department of Cell and Molecular Biology, University of Hawaii, 1960 East–west Rd, Honolulu, HI 96822 USA; State Institute of Fisheries Technology, Jagannayak Pura, Kakinada, Andhra Pradesh India; Human Nutrition, Food & Animal Science, College of Tropical Agriculture & Human Resources (CTAHR), University of Hawaii At Manoa, 1955 East West Road, AgSci 216, Honolulu, HI 96822 USA; Paul Hebert Centre for DNA Barcoding and Biodiversity Studies, Dr. Babasaheb Ambedkar Marathwada University, Aurangabad, 431004 India

**Keywords:** *Penaeus monodon*, Population genetics, Hatchery, Mt DNA, Dloop, Diversity

## Abstract

Quality production of the shrimp *Penaeus monodon* in hatchery operations depends heavily on the evaluation of genetic diversity and population structure of brood stocks*.* Mitochondrial DNA (mtDNA) sequences have been widely used to study genetic variability and relationships in many crustacean groups, and these same markers may be incorporated into evaluation studies of shrimp broods and populations. For this purpose we looked at variation in mitochondrial D-loop sequences as an indicator of genetic diversity in shrimp populations from a region of India that represents the main sources of new material for brood stocks. In our study of these populations the overall mean genetic diversity was 0.191. The highest level of genetic diversity (0.357) was observed in the Kakinada population, whereas the lowest diversity (0.0171) was observed in the Nellore population. The results also indicate that overall, the populations along the Andhra Pradesh coast are genetically diverse despite the fact that there is considerable gene flow between them. From the results, it is evident that east cost of India shows high genetic diversity among *P. monodon* broods and no evidence of loss of diversity due to excessive inbreeding. The fact that the genetic variability of these populations has been maintained, despite ten years of dependence on these broods, shows that at the present time there is no indication of over exploitation.

**Electronic supplementary material:**

The online version of this article (doi:10.1186/2193-1801-2-671) contains supplementary material, which is available to authorized users.

## Introduction

The genus *Penaeus* represents an economically important group of shrimps and prawns (Dall et al. 1990; Bailey-Brook and Mass 1992; Rosenberry 2001). Aquaculturing of *Penaeus monodon* alone accounts for more than 50% of the world’s cultured shrimp (Ronnback 2001). However, because of limited reproductive capacity in captivity, continued culturing is highly dependent on wild caught brood stocks (Spann et al. 1997). Also, in hatchery operations, the identification and evaluation of comparative growth performance of existing stocks is necessary (Benzie 1994). Hence, basic knowledge about genetic markers, levels of genetic diversity and differentiation in broods and populations is imperative for construction of an appropriate genetic based stock enhancement programme and to identify regions that may be over exploited and where artificial recruitment may be required (Kumar et al. 2007).

Mitochondrial DNA (mtDNA) sequences are widely used to study genetic variability in aquaculture species including crustaceans, and these sequences have proved extremely useful in elucidating genetic variability and phylogenetic relationships among many crustacean groups (Cunningham et al. 1992; Chu et al. 2003). These regions may also contain ideal markers for characterizing geographical patterns of genetic variation within and between prawn populations (Simon 1991). The complete mitochondrial genome of *P. monodon* is around 16 kb (Wilson et al. 2000), of which 991 bp is the long noncoding, ‘AT’ rich control region known as the D-Loop. This region plays a significant role in mitochondrial replication and DNA transcription, and it contains the signals that control many general aspects of RNA and DNA synthesis. Previous reports employing mtDNA D-loop based studies on penaeids have demonstrated the usefulness of this region in genetic variability studies (Chu et al. 2003; Tzeng et al. 2004 and Kumar et al. 2007).

Domestication of *P. monodon* has been carried out for production of high-quality pond-reared *P. monodon* brood stocks (Withyachumnarnkul et al. 1998), but the program recently collapsed from a white spot syndrome virus (WSSV) infection. Identification of genetically diverse and geographically differentiated shrimp stocks will be essential for both re-establishing and maintaining effective domestication and breeding programs for *P. monodon*. In addition, over exploitation of *P. monodon* may be avoided by continuous monitoring and possible enhancement through the use of natural populations (Klinbunga et al. 2001).

Along the coast line of the area known as Andhra Pradesh (974 Km), the dominant shrimp culture area in India, shrimp hatcheries are clustered in three areas: Vizag (North Andhra), Kakinada (Central Andhra) and Nellore (South Andhra). Out of 280 *P. monodon* hatcheries in the country, 148 are located in this region (Andhra Pradesh). These produce 7882 million larvae per year, and this represents approximately 63% of total seed production in India. Keeping in mind the importance of shrimp culture to the economy and the degree to which success is primarily dependent on the health of the seed and the brood stock, the present work has been conducted to study the genetic structure and diversity of brood stocks from the Andhra Pradesh (A.P.) area in India to ensure the ability to maintain genetically diverse brood stocks for improved production.

## Materials and methods

### Sample collection

**Figure 1:**
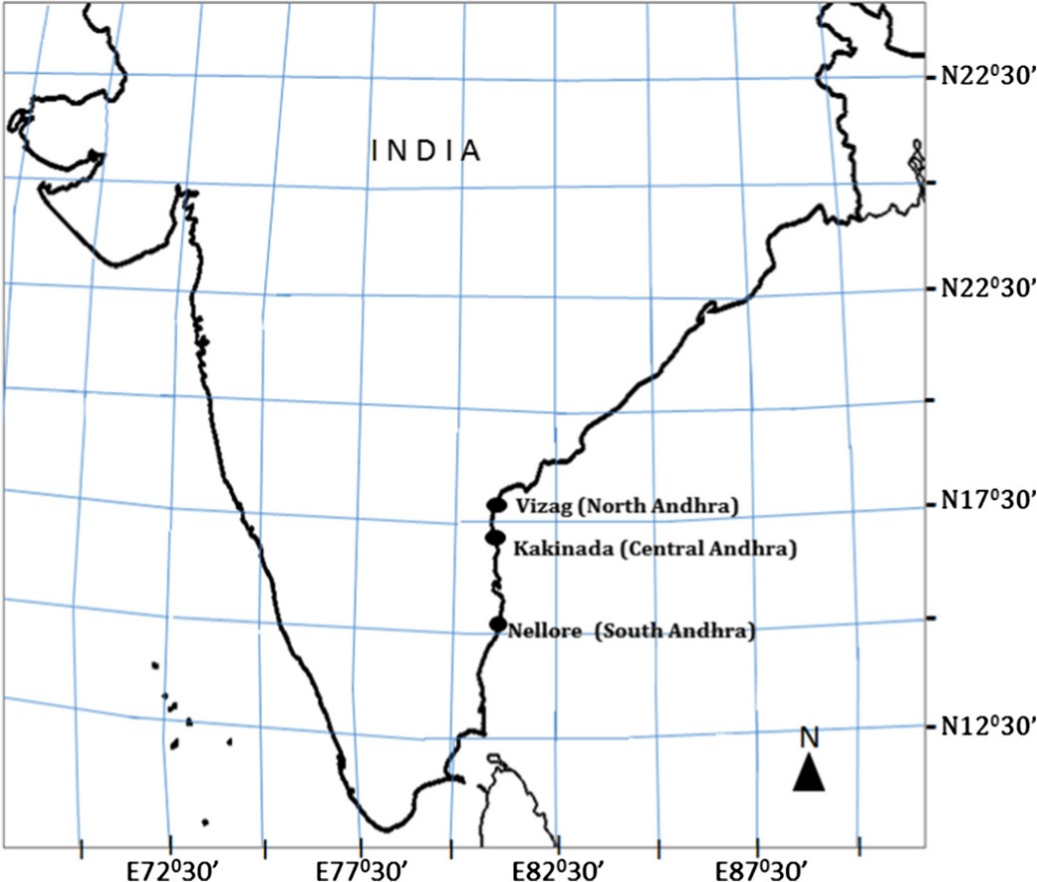
**Sampling locations along the East Coast of India.**

Wild samples of *P. monodon* brooders were collected from the three regions along the A.P. coast (Figure 1). The study area was demarcated into three regions as Vizag (North Andhra), Kakinada (Central Andhra) & Nellore (South Andhra), where the shrimp hatcheries are clustered. The shrimp broods were collected (30 individuals per population) during January- February, 2011 using mechanised boats having bottom trawl nets specifically designed for shrimp catch. Shrimp caught were washed with clean water and pleopods were dissected and preserved in 95% ethanol at −4°C until they could be further processed.

### Genomic DNA isolation

Genomic DNA was isolated from pleopods following the method described by Sambrook et al. (2005), and the DNA was diluted to obtain a final concentration of 100 ng/μl.

### PCR primers

The primers used here for PCR amplification are 12S (F) -5′ AAGAACCAGCTAGGATAAAACTTT 3′ and 1R (R) 5′-GATCAAAGAACATTCTTTAACTAC-3′. These were derived from Chu et al. (2003) and Yang et al. (2006).

### PCR amplification reactions

The mtDNA control region was amplified in a 25 μl reaction volume with a final concentration of 1X Taq polymerase buffer and 0.6 U of Taq polymerase, 1.5 mM MgCl_2_, 2.5 mM dNTPs and 1.5 μM each primer. The thermal profile for hot-start PCR included initial denaturation at 95°C for 5 min, followed by 35 cycles of 20 s at 94°C, 30 s at 48°C, 60 s at 68°C and a final extension of 10 min at 68°C. The PCR product was purified by treatment with exonuclease and shrimp alkaline phosphatase at 37°C for 30 min, and the enzyme inactivation was carried out at 85°C for 15 min. Products were cleaned by ethanol precipitation and sequenced using an ABI Prism DNA analyzer 3730 (Applied Biosystems, USA) and the Big dye cycle sequencing kit.

### Data analysis

A total of 81 sequences, each 562 bp in length (on average) from the mtDNA control region, were obtained for analysis. Nine of the original samples with incomplete sequence reads were not included in analysis. The usable sequences were aligned using Bio-edit sequence editor package (Hall 1999), and data analysis was performed using ARLEQUIN version 3.0 (Excoffier et al. 2005) and MEGA 4. The mean nucleotide composition, number of transitions, transversions, indels, number of haplotypes, haplotype diversity (h) and nucleotide diversity (*pi*) values (Nei 1987) were calculated for all the populations. The haplotype data were analysed phylogenetically by the neighbour-joining (NJ) method using MEGA 5.0 and the genetic distance by the Jukes and Cantor (1969). Support for the tree nodes was assessed by the bootstrap method (1000 replicates). The geographical structuring of population was examined by performing analysis of molecular variance (AMOVA) to partition the total genetic variation into its variance component and to produce F_ST_ statistics (Weir and Cockerham 1984).

## Results

### Genetic diversity and lineages

The partial mitochondrial control region (D-Loop) sequences of 562 bp in length (average) from 81 individuals (NCBI accession nos. JQ863127 to JQ863216) analyzed here show 43 haplotypes with a value of 14.33 for mean haplotype diversity. The overall Jukes-Cantor estimate of nucleotide diversity (*pi*) for all of the samples analyzed here is 0.452 ± 0.1415, with a mean value for the populations of 0.150 ± 0.141. The average number of differences 18.8443 and the total number of segregating sites is 417 (Table 1).

**Table 1:** **D-loop sequence based diversity analysis**

	No. of seq.	NCBI accession numbers	No. of seg. sites S	No. of Haplotype	Haplotype diversity Hd	No. of polymorphic loci	Expected heterogenity	Total no. of alleles	Avg. no. of differences K	Avg. no, of pairwise differences	Nucleotide diversity with JC ***Pi***-JC
Vizag	27	JQ863127-JQ863156	143	14	0.9195	38	0.03345	1.236	11.0022	6.386	0.035086 ± 0.01910
Kakinada	24	JQ863157-JQ863186	251	21	0.9310	129	0.15094	1.934	40.1207	37.215	0.204479 ± 0.102747
Nellore	30	JQ863187-JQ863216	23	8	0.8643	47	0.03468	1.165	4.8392	6.635	0.036457 ± 0.019701
Total	**81**		**417**	**43**	**2.7148**		**0.07546**	**2.192**	**90.4458**	**0.3162**	**0.4522** ± **0.1415**
		**Mean**	**139**	**14.33**	**0.9049**	**71.333**			**18.8443**	**17.727**	**0.1507** ± **0.0471**

The Kakinada population contained the largest number of haplotypes (21) and the highest value overall for haplotype diversity (0.931). This was closely followed by the Vizag population where 14 haplotypes produced a diversity value of 0.9195 and finally the Nellore population which had 8 haplotypes and a value for haplotype diversity of 0.864. Most of the haplotypes identified here (42 out of 43) were unique to one of these populations. The one shared haplotype was found in all three of the populations studied here.

Other parameters measuring variation among the three populations are shown in Table 1. A mean expected heterogeneity value of 0.075 was observed among three populations. The individual values ranged from a high of 0.150 in Kakinada to a low of 0.033 in the Vizag population (Table 1). The overall Jukes Cantor (Pi-JC) nucleotide diversity at Vizag population was 0.0350, Kakinada 0.204 and Nellore was 0.036. The average number of pair wise differences (k) is 17.727, with the highest number of differences observed in the Kakinada population which was 37.215 and lowest in Vizag (6.386).

### Population structure

For the three populations a total of 417 segregating sites were observed. Also for these populations overall mean numbers of 38 (transition) and 43 (transversion) type substitution mutations were observed. The Kakinada population had the highest numbers of both transitions (74) and transversions (96) whereas lower numbers (22 and 8, respectively) were seen in the Nellore population. An overall average of 81 substitutions were noted with the least number occuring in the Nellore population (30), followed by 43 in Vizag and the highest in the Kakinada region (170). Indels were absent in all three populations (Table 2).

**Table 2:** **Transition/transversion values**

Statistics	Vizag	Kakinada	Nellore	Mean	S.D.
No. of transitions	18	74	22	38.000	31.241
No. of transversions	25	96	8	43.000	46.680
No. of substitutions	43	170	30	81.000	77.350

The overall proportions of nucleotides in this dataset are 0.395 (A), 0.409 (T/U), 0.108 (C), and 0.088 (G) based on a total of 286 positions. The transition/transversion rate ratios are *k*_*1*_ = 24.707 (purines) and *k*_*2*_ = 31.233 (pyrimidines). The overall transition/transversion bias is *R* = 5.623, where *R* = [A*G*k_*1*_ + T*C*k_*2*_]/[(A + G)*(T + C)]. All positions containing gaps and missing data were eliminated.

### Population level variation

An Analysis of Molecular Variance (AMOVA) was performed in MEGA5.0 for 81 sequences to test for geographic variations/divisions among populations. Results from this analysis showed a percentage of variation attributable to among-population differences of 11.04% whereas most of the variation (88.96%) was attributed to variation within populations (Table 3).

**Table 3:** **AMOVA analysis**

Source of variation	d. f.	Sum of square	Variance components	Percentage of variation
Among population	2	468.44	6.154	11.04
within populations	87	4312.83	49.57	88.96

The pairwise Fst comparisons in Table 4 show that the Vizag and Nellore populations are the most differentiated whereas the Vizag and Kakinada populations are the least differentiated. Estimates of Nm to reflect gene flow between populations are given in Table 5. The Nm values between the populations of Vizag and Nellore were relatively lower (3.0486) compared to the highest value of 4.775 seen between the Vizag and Kakinada populations. All of these values, however, are greater than 1.

**Table 4:** **Population pairwise FSTs**

	Vizag	Kakinada	Nellore
Vizag	0.0000		
Kakinada	0.05309	0.00000	
Nellore	0.21819	0.09555	0.00000

**Table 5:** **Nm values between populations**

Gene flow (Nm) between populations	
Vizag and Kakinada	4.775
Vizag and Nellore	3.048
Kakinada and Nellore	3.966

**Figure 2:**
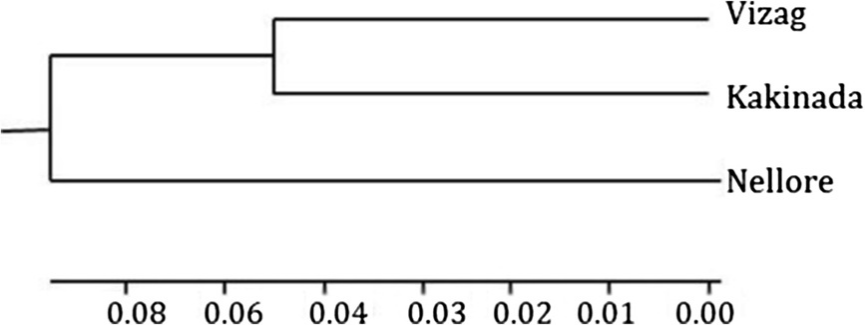
**Neighbor joining tree for populations.**

A Neighbour Joining (NJ) tree was constructed which depicts the overall relationships of the populations studied here (Figure 2). This tree is divided into two lineages. One contains the Nellore population alone while the second lineage contains both the Vizag and Kakinada populations. This overall lineage relationship is also supported by the Neighbor Joining tree run for all individuals (Additional file 1: Figure S1).

## Discussion

Polymorphisms in mtDNA sequences have been used previously for examining both intraspecific population differentiation and phylogenetic relationships of some penaeid shrimp populations (Benzie 2000; Lavery et al. 2004). This study reports the analysis of genetic variation in the mtDNA D-loop region of *P. monodon,* a commercially important shrimp species (Bailey-Brook and Mass 1992; Rosenberry 2001), and provides information about the genetic structure and relationships of populations from a region which accounts for the majority (63%) of brood seed production in India (AISHA-All India Shrimp Hatcheries Association 2004; FAO 2004;2006). Because this is the largest shrimp producing and seed supplying region in India, a major goal of this study was to provide baseline data for estimates of genetic diversity and population structure of *P. monodon*. Analysis of genetic variability and geographic differentiation of such organisms is essential for the development of effective resource management programs (Avise 1994). This type of information is required for maintaining and improving the culture and management efficiency of *P. monodon* (Carvalho and Hauser 1994; Ward and Grew 1994). In general, relatively low degrees of genetic differentiation have been seen in wild *P. monodon*, even for those separated over distances of hundreds or thousands of kilometres, except where major biogeographic boundaries act to disrupt gene flow (Benzie et al. 2002).

Among the regions studied here, high levels of mtDNA diversity were observed overall. This is generally consistent with findings from previous studies for decapods and penaeids in general (Silberman et al. 1994; Baldwin et al. 1998; Benzie et al. 2002) although the overall nucleotide and haplotype diversity values obtained in this study are among the highest reported (0.1507 and 0.9049 respectively) for this species. Previously the maximum haplotype diversity reported for *P. monodon* was 0.682 ± 0.002 (Benzie 2000) and the maximum nucleotide diversity was 0.00334 ± 0.00003 (Klinbunga et al. 1998). We obtained haplotype diversity values of 0.9195, 0.9310 and 0.8634 for the Vizag, Kakinada and Nellore populations, respectively. The genetic diversity for the Kakinada population also appears to be greater compared to that of the other populations. The values we obtained for these *P. monodon* populations are, however, comparable with those derived from mtDNA d-loop region sequences of the black shrimp *Caridina cantonesis*, the white shrimp *Panaeus setiferus*, and the pink shrimp *Farfantepenaeus duorarum*, (McMillen-Jackson and Bert 2003,2004; Kumar et al. 2007 and Khamnamtong et al. 2009).

The AMOVA results show that most of the variation (88.96%) detected here is found within populations. Our results also suggest that overall, high levels of gene flow (as reflected by Nm values) are occuring between these populations. Nevertheless, as indicated by the pairwise Fst values, the mixing of lineages in *P. monodon* in India has clearly not been complete. This could be explained by some ecological or environmental factors such as major physical barriers, pollution or reversals in the monsoon-driven surface water current systems (Dale 1956). Similar findings were reported by Khamnamtong et al. (2009) and Mandal et al. (2012). Also in Australia (Benzie et al. 2002), low levels of population genetic differentiation in wild *P. monodon* were evident over distances of hundreds or thousands of kilometers, except where major biogeographical boundaries acted to disrupt gene flow.

The NJ trees constructed using control region sequence data also showed a general population structuring according to geographical distribution. However, a number of mixed lineage hapolotypes were found at present in each geographic sample, reflecting some secondary mixing of those haplotypes. This may be explained by the fact that the spawning behavior of *P. monodon* females can enhance levels of lineage mixing because they migrate offshore when they grow and mature (Motoh 1981). Regardless, the overall relationships shown by the NJ trees that group the Vizag and Kakinada populations together is again consistent with the apparent high levels of gene flow and relatively low levels of genetic differentiation seen between these two populations as compared to the Nellore populaton.

The levels of genetic diversity revealed in the present study using this mtDNA control region might be useful as genetic indicators for aquaculture purposes including planning for selective breeding, maintaining stock diversity and distinguishing hatchery stocks from the wild populations. Some of this diversity may be explained by a high rate of mtDNA mutation as has been suggested for several other penaeid species (Palumbi and Benzie 1991; Baldwin et al. 1998). The basic knowledge of genetic divergence between evolutionary lineages, and the existence of population differentiation between major stocks of Indian *P. monodon*, suggests that each population should be treated as a separate management unit because it may display unique demographic and dynamic properties (Carvalho and Hauser 1994; Conover et al. 2006).

The assessment of genetic diversity and population structure of *P. monodon* is critical for appropriate conservation and management purposes. With increased farming and opportunities for future growth in the aquaculture of *P. monodon*, there is a great concern regarding the loss of wild genetic diversity. For good production, hatchery operators often collect brooders from different parts of the country (AISHA-All India Shrimp Hatcheries Association 2004 and FAO 2006). Similar observations were made by Klinbunga et al. (1998) in Thailand where farmers believe that progeny of the Andaman Sea *P. monodon* exhibit greater survival and possibly greater growth rates than do progeny from broodstock shrimp caught elsewhere in Thailand*.* Therefore, genetic monitoring and evaluation of black tiger shrimp can help to identify any negative effects on genetic diversity caused by aquaculture (Naylor et al. 2000; Benzie 2010). Also, maintaining high levels of genetic diversity and population differentiation of *P. monodon* can help to protect this species from disease epidemics and severe population declines. This would further facilitate the stock improvement programme of this commercially important species through selective breeding. The virtual absence of domesticated specific pathogen free stocks of *P. monodon* has inhibited breeding programme development and commercial production of this species (Clifford and Preston 2001). Sourcing and spawning of clean founder stocks from wild populations is one means to generate domesticated pathogen free stocks of *P. monodon*. It is widely accepted that the most economically significant viral pathogens like WSSV, yellow head virus and a host of other pathogens have been introduced into the Asian countries through the careless introduction of live shrimp stocks. Import of disease-free stocks from these regions of India or elsewhere will be beneficial when stocks are used that are free from these and other pathogens and/or viruses.

## Conclusions

Information about genetic variability of critical populations and the potential for improvement using biotechnological applications are crucial for the maintenance and future development of shrimp industry. A high level of genetic diversity has been revealed in the present study using the mtDNA control region. The nucleotide and haplotype diversities obtained in this study are among the highest reported for *P. monodon* populations. The genetic diversity at Kakinada appears to be greater than that of Vizag and Nellore. The relatively high Fst values seen for all of these populations, together with the fact that most of the variation detected here occurs within populations, also indicate that in this region, this species is genetically heterogenous and does not appear to be suffering from extensive inbreeding. The genetic diversity seen here suggests that farmers or hatchery operators can continue to use these populations as sources of natural broodstock from this region of India. Finally, the information obtained here may also be useful for providing genetic markers that can be used for aquaculture purposes such as planning for selective breeding, maintaining stock diversity and distinguishing hatchery stocks from the wild populations.

## Electronic supplementary material

Additional file 1: Figure S1: NJ Phylogenetic tree of all individuals from three populations. (DOCX 326 KB)

Below are the links to the authors’ original submitted files for images.

Authors’ original file for figure 1

Authors’ original file for figure 2
