# Supplementary figures and images for: High levels of genetic diversity in Penaeus monodon populations from the east coast of India
Source: Springerplus. 2013 Dec 13;2(1):671. doi: 10.1186/2193-1801-2-671 (PMC3868705; doi:10.1186/2193-1801-2-671)

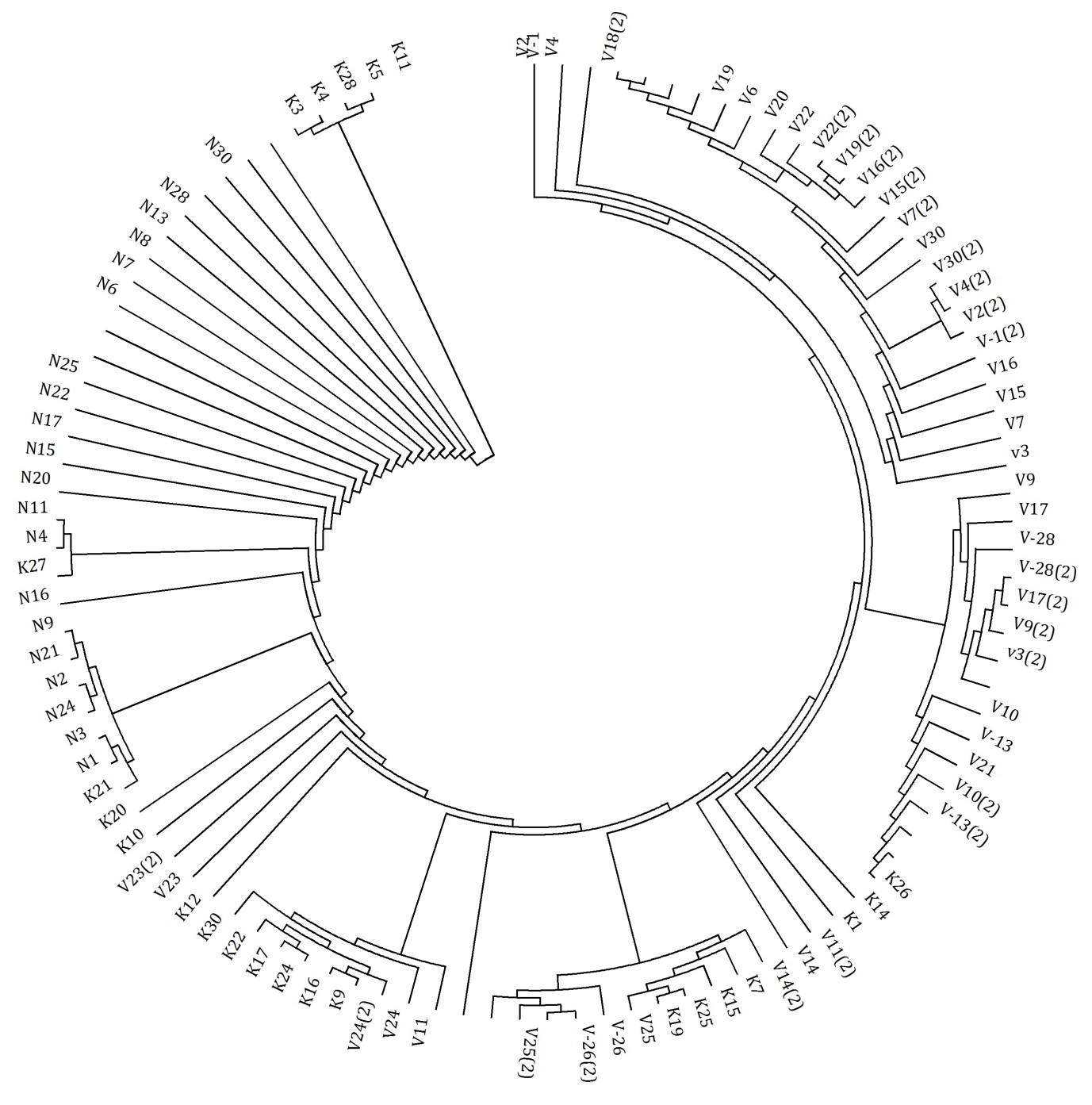


**Supporting Fig. 1** NJ Phylogenetic tree of all individuals from three populations

Supplement: Supplementary file 1 — Additional file 1: Figure S1: NJ Phylogenetic tree of all individuals from three populations. (DOCX 326 KB) [file 40064_2013_736_MOESM1_ESM.docx]

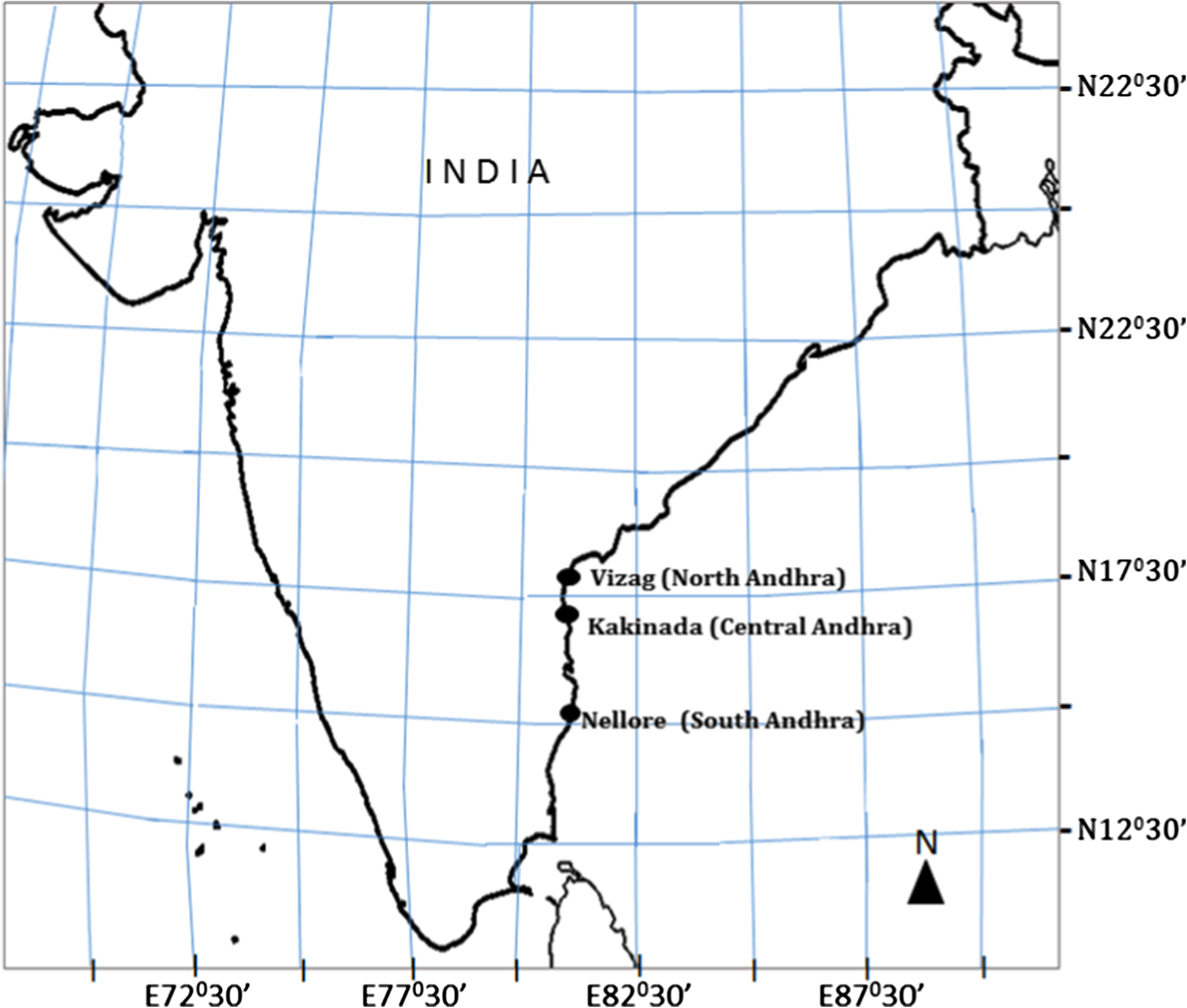

Supplement: Supplementary file 2 — Authors’ original file for figure 1 [file 40064_2013_736_MOESM2_ESM.tif]

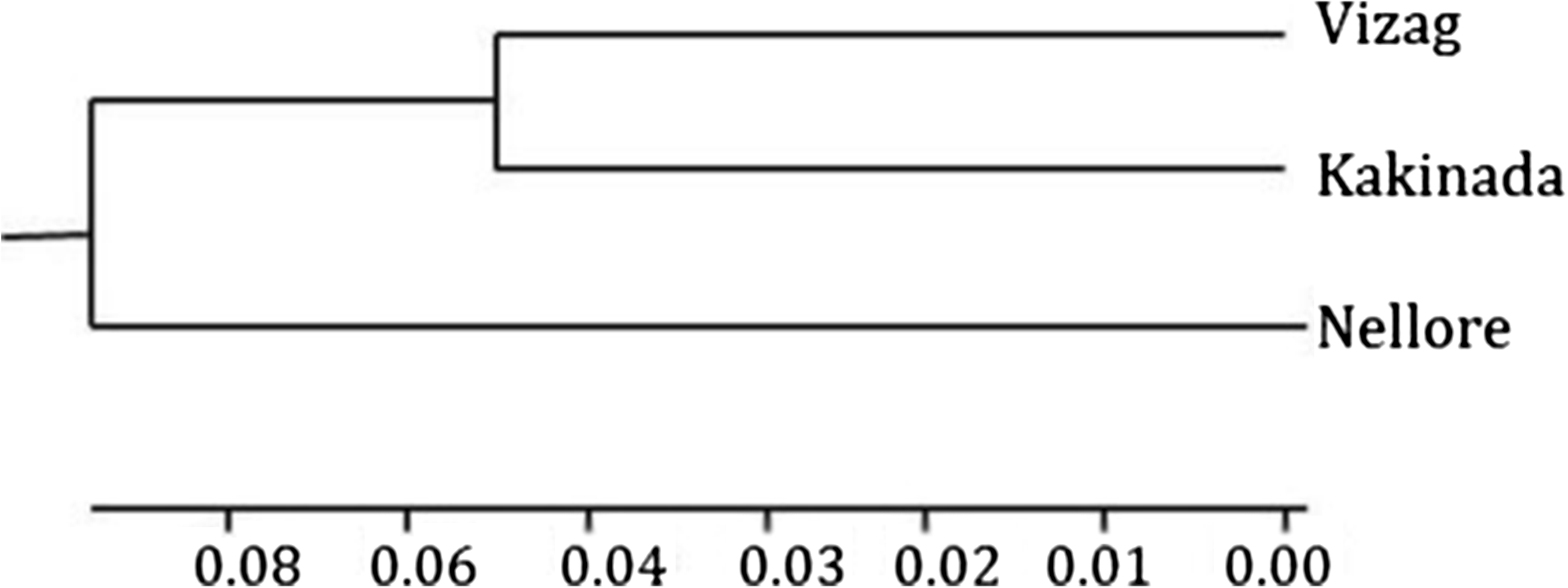

Supplement: Supplementary file 3 — Authors’ original file for figure 2 [file 40064_2013_736_MOESM3_ESM.tif]
